# Nematicidal Efficacy of Milbemectin against Root-Knot Nematodes

**DOI:** 10.3390/plants9070839

**Published:** 2020-07-03

**Authors:** Miguel Talavera-Rubia, Maria Dolores Vela-Delgado, Soledad Verdejo-Lucas

**Affiliations:** 1 Institute for Research and Training in Agriculture and Fisheries, IFAPA Alameda del Obispo, Av. Menéndez Pidal s/n, 14004 Córdoba, Spain; 2 Institute for Research and Training in Agriculture and Fisheries, IFAPA Rancho de la Merced, Crtra, CA-3102, Km, 3.1, 11471 Jerez de la Frontera, Cádiz, Spain; mdolores.vela@juntadeandalucia.es; 3 Institute for Research and Training in Agriculture and Fisheries, IFAPA La Mojonera, Autovía del Mediterráneo, salida 420, Paraje San Nicolás, 04745 La Mojonera, Almería, Spain; soledadverdejo@gmail.com

**Keywords:** Biopesticide, Nematicide, *Meloidogyne*, *Solanum lycopersicum*

## Abstract

The nematicidal efficacy of milbemectin and its commercial formulate Milbeknock^®^ on (i) egg hatching, (ii) juvenile motility and (iii) infective capacity of root-knot nematodes was evaluated in vitro and in planta assays. Serial dilutions of pure milbemectin were tested against nematode eggs and juveniles and lethal concentrations LC50 and LC90 calculated. Exposure of egg masses to milbemectin at a concentration of 30 μg/mL for 72 h reduced egg hatching by 52%. The increase in exposure time to 240 h did not increase the egg hatching inhibition at the highest concentration 30 μg/mL (53%) but reduced egg hatching at 15 and 7 μg/mL by 35 and 24%, respectively, when compared to untreated controls. The inhibitory effect of milbemectin on juvenile motility ranged from 41 to 87% depending on its concentration, and this effect was persistent after rinsing the juveniles in water. The probabilistic dose–response model indicated that lethal concentrations of milbemectin for juvenile motility were LC50: 7.4 μg/mL and LC90: 29.9 μg/mL. The pre-plant application of Milbeknock^®^ to soils infested with the nematode reduced its infective capacity by 98–99% compared to untreated soils in pot experiments. Milbeknock^®^ reduced nematode soil population densities by 50–60% in natural infestations under field conditions. Milbemectin shows a high level of efficacy against root-knot nematodes as it reduces egg hatching, persistently immobilizes nematode juveniles, and reduces tomato root infection.

## 1. Introduction

Plant parasitic nematodes of the genus *Meloidogyne* (root-knot nematodes, RKN) are important plant pathogens difficult to control due to their wide host range and huge reproductive potential. Conventionally, RKN control has been performed through soil chemical fumigation, but bans and restrictions on the use of most chemical fumigants, such as methyl bromide, 1.3-dichloropropene, chloropicrin or metam-sodium have brought serious concerns regarding control of plant parasitic nematodes in intensive horticulture. Currently, five active ingredients are authorized as nematicides for use in vegetable crops in Spain: abamectin, fenamiphos, fosthiazate, fluopyram and oxamyl. The frequent use of these nematicides could exert high selective pressure on RKN and soil microbiota. For this reason, the search for new efficient nematicides with different modes of action to the long-time used organophosphate and carbamate nematicides is presently a priority in nematological research.

The search and use of natural pesticides (biopesticides) are promoted by environmental and plant health authorities in Europe. Biopesticides are chemicals derived from natural materials as animals, plants, bacteria, and certain minerals or microorganisms themselves with suppressive effects on plant pathogens and pests. Biopesticides have various advantages over the synthetic chemicals use as phytosanitary products; (i) they are usually less toxic for humans than conventional pesticides; ii) they have a narrower spectrum of action than conventional pesticides, which frequently affect non-target organisms; (iii) they often decompose quickly, resulting in lower exposures and largely avoiding the pollution problems caused by conventional pesticides.

Milbemectin is an antibiotic isolated from the soil bacterium *Streptomyces hygroscopicus* subsp. *aureolacrimosus*, which shows acaricidal activity against spider mites and nematicidal activity against *Caenorhabditis elegans* (LC50: 9.5 μg/mL) [1], *Bursaphelenchus xylophilus* (LC20: 0.1 μg/mL) [2] and *Hirschmanniella diversa* [3]. Furthermore, milbemectin is a macrocyclic lactone with similar molecular structure to other members of the family used as biopesticides against RKN, as abamectin, ivermectin and emamectin [4,5,6,7,8]. Therefore, some nematicidal activity of milbemectin would be expected, but to our current knowledge, no information is available on its potential use as a nematicide.

The main objective of this study was to determine the effects of pure milbemectin and a commercial formulation of milbemectin, Milbeknock^®^ on RKN: (i) egg hatching; (ii) juvenile motility, (iii) root infection, (iv) nematode reproduction and (v) RKN symptoms on tomato plants.

## 2. Results

### 2.1. Effects of Milbemectin on Egg Hatching

**Figure 1:**
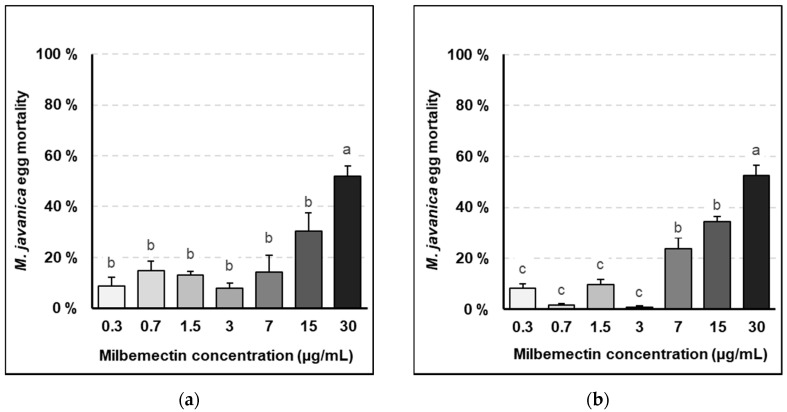
*Meloidogyne javanica* egg mortality due to progressively higher concentrations of pure milbemectin. (**a**) After 72-hour exposure; (**b**) after 240-h exposure to milbemectin. Data are mean of eight replicates. Error bars indicate the standard error of the means. Bars with the same letter do not differ significantly by the HSD Tukey test (*p* < 0.05).

Egg hatching was reduced by the milbemectin 30 μg/mL concentration after 72 h exposure (*p* < 0.05), which represented a 52% inhibition over the untreated control (Figure 1a). Lower concentrations of milbemectin did not affect egg hatching after 72 h exposure (*p* > 0.05). However, after 240 h exposure, milbemectin concentrations 7 and 15 μg/mL did reduce egg hatching by 24 and 35%, respectively, though the highest inhibitory effect was also observed at the highest concentration (30 μg/mL), with a 53% inhibition over the untreated control (Figure 1b).

**Figure 2:**
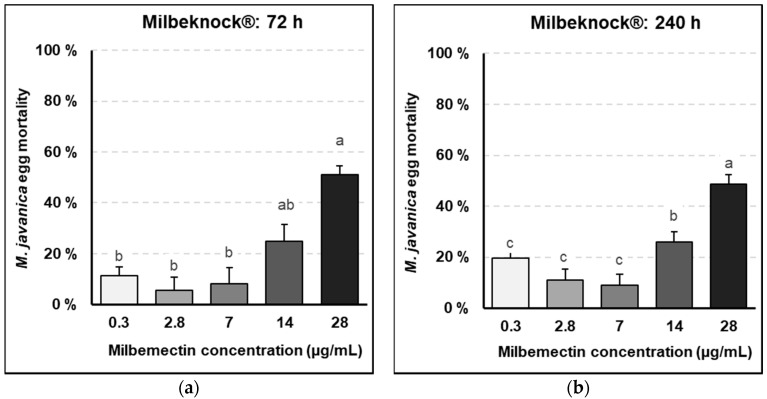
*Meloidogyne javanica* egg mortality due to progressively higher concentrations of Milbeknock^®^. (**a**) After 72-h exposure; (**b**) after 240-h exposure to Milbeknock^®^. Data are mean of eight replicates. Error bars indicate the standard error of the means. Bars with the same letter do not differ significantly by the HSD Tukey test (*p* < 0.05).

Similar values were obtained with the commercial formulate Milbeknock^®^, with a 51% of egg hatching inhibition over the untreated control at the highest milbemectin concentration (28 μg/mL) after 72 h exposure (*p* < 0.05) (Figure 2a) and 49 and 26% egg hatching inhibition at the 28 and 14 μg/mL concentrations, respectively after 240 exposure (*p* < 0.05) (Figure 2b).

These reductions in egg hatching did not persist after rinsing the eggs in water, since 42–57% of the non-hatched eggs, hatched after 48 h in tap water, with no differences among concentrations of milbemectin (*p* > 0.05).

Lethal doses of milbemectin, which inhibited nematode egg hatching in vitro by 50 and 90% were LC50: 30.3 μg/mL and LC90: 57.8 μg/mL, after 72 h exposure and LC50: 28.9 μg/mL and LC90: 50.9 μg/mL, after 240 h exposure.

### 2.2. Effects of Milbemectin on Juvenile Motility

**Figure 3:**
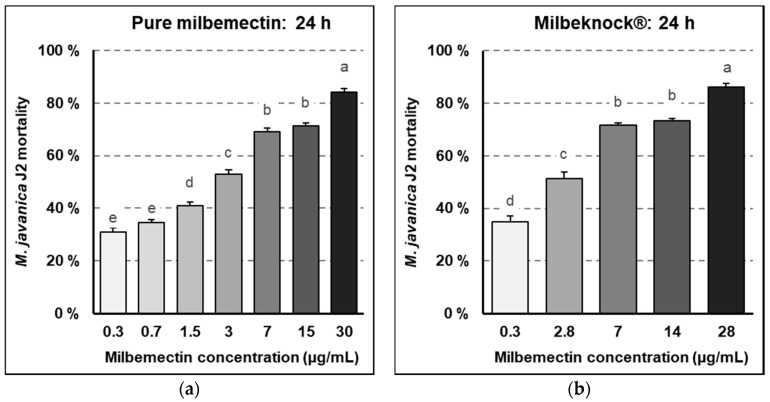
Mortality of *Meloidogyne javanica* second-stage juveniles due to progressively higher concentrations of pure milbemectin and Milbeknock^®^. (**a**) After 24-h exposure to milbemectin; (**b**) after 24-h exposure to Milbeknock^®^. Data are mean of eight replicates. Error bars indicate the standard error of the means. Bars with the same letter do not differ significantly by the HSD Tukey test (*p* < 0.05).

Milbemectin inhibited the motility of RKN juveniles at all concentrations by 31 to 84%, over untreated controls, after 24 h exposure. The reduction in motility was proportional to the milbemectin concentration, being highest at 30 μg/mL (Figure 3a). Similar values were obtained with the commercial formulate Milbeknock^®^, with a 86% inhibition of J2 (juveniles of stage 2) motility over the untreated control, at the highest concentration (28 μg/mL), and 35% at the lowest concentration (0.3 μg/mL), after 24 h exposure (*p* < 0.05) (Figure 3b).

The inhibitory effect on nematode motility was highly persistent since the percentage of juveniles recovering their motility was lower than 2% at all concentrations after incubation in water.

The dose–response probabilistic model (PROBIT) for juvenile motility indicated that LC50 was 7.4 μg/mL whereas the LC90 was 29.9 μg/mL.

### 2.3. Effects of Milbeknock^®^ on Nematode Root Infection

The nematode infected tomato roots in the RKN-infested pots and completed a generation cycle, as shown by the presence of galls with egg masses in the roots. Few egg masses were observed in the Milbeknock^®^ treated plants, which indicates that nematodes escaping the action of the milbemectin were able to infect and develop on tomato. However, the application of 0.3 mL of Milbeknock^®^ (2.8 mg of milbemectin) per pot significantly reduced the infection capacity of the nematode (*p* < 0.05), and such reduction ranged from 98 to 99% in relation to the untreated nematode-infected plants (Table 1).

Tomato growth parameters in the pot experiments are shown in Table 2. Milbeknock^®^ treated and untreated plants did not differ in plant growth parameters in the presence or absence of the nematode after one generation cycle.

### 2.4. Effects of Milbeknock^®^ on Nematode Disease on Tomato in Field Trials

No significant differences between elementary plots were observed for *Meloidogyne incognita* soil densities prior to treatments (P0) (*p* > 0.05) and, thus, the homogeneity of *M. incognita* soil populations between the plots at the start of the test was assumed. Average *M. incognita* soil population densities were 138 ± 15 and 154 ± 27 J2 per 250 g of soil, in field trials 1 and 2, respectively. All treatments reduced *M. incognita* soil densities at planting (Pi) compared to the untreated control (*p* < 0.05). Milbeknock^®^ at 2.5 L/ha reduced *M. incognita* soil populations by 59% and it was equally effective than Tervigo^®^ at 5 L/ha (61%). However, Milbeknock^®^ at 1.5 L/ha was less (*p* < 0.05) effective in reducing nematode soil densities (52%) (Table 3). The severity of the disease caused by *M. incognita*, estimated by root galling indices, ranged from 4.6 to 5.5 with no differences between treatments (*p* > 0.05) (Table 3).

Regarding tomato yield, higher number of fruits were collected from plots treated with Tervigo^®^ than untreated control and treatments with Milbeknock^®^ (*p* < 0.05) (Table 4). Milbeknock^®^ treatments did not differ from the untreated control in fruits numbers. Total fruit weight did not differ between treated and untreated plots (*p* > 0.05) under the experimental conditions (Table 4).

## 3. Discussion

Milbemectin killed RKN J2, inhibited egg hatching and reduced nematode infection capacity in vitro, in planta and in field trials. Milbemectin immobilized 50% and 90% of the J2 permanently at concentrations of 7.4 and 29.9 μg/mL, respectively since motility was not recovered after rinsing J2 in water. Therefore, we consider that the increased mortality was causally related to increasing concentrations of milbemectin. The LC50 and LC90 values affecting egg hatching and J2 motility for the pure milbemectin and the commercial formulate Milbeknock^®^ were very close (Figure 1 and Figure 2), which indicates that the excipients of the formulated product Milbeknock^®^ did not interfere with the nematicidal activity of the active ingredient. The toxicity values of milbemectin to the nematode were similar to those reported for emamectin (LC50: 3.6 μg/mL; LC90: 18.2 μg/mL) [8] and abamectin (LC50: 1.7–4.8 μg/mL; LC90: 4.7–25.7 μg/mL) [9]. The similarities in toxicity values of milbemectin, abamectin and emamectin suggest that these macrocyclic lactones share a similar mode-of-action against *Meloidogyne* species. The mode-of-action of milbemectin is presently unknown but that of abamectin and emamectin is associated with their effect on the δ-aminobutyric acid (GABA) receptors and glutamate-gated chloride channels, increasing the permeability of chloride ions, hyperpolarizing the nerve and muscle cells, and disturbing the neuromuscular transmission leading to death [10].

Milbemectin also inhibited egg hatching by 50% at a concentration of 30 μg/mL, after 72 h exposure, but this inhibition can be related to the paralyzed J2 within the eggs that could not break the eggshell and hatch, since movement and thrusting of the stylet is an integral part of the emergence from eggs in most plant parasitic nematodes. However, this effect was not persistent since 42–57% of the eggs hatched once rinsed in water.

Reductions in the infection capacity of RKN on tomato were confirmed in pots, since milbemectin treated plants showed up to 99% reduction in the number of RKN egg masses when compared to untreated nematode-infected plants. Numbers of RKN galls and egg masses on tomato were also reduced 66% by abamectin applications [11]. Drenching the pot soil with a high concentration of Milbeknock^®^ (equivalent to 2 kg/ha) maximized the efficacy of the product in nematode control as root infection was reduced by 99%. The volume of water used for the application allowed a good distribution of the product because it wetted the entire soil profile.

The effectiveness of milbemectin in reducing RKN soil densities in field trials ranged between 50–60%. Similar efficacy levels have been reported for currently registered non-fumigant nematicides in Spain against RKN [12,13]. Increasing the rate of Milbeknock^®^ from 1.5 to 2.5 L/ha did not significantly increase the efficacy of the product under test conditions. Despite similar LC values for the three lactones, field studies have demonstrated that different doses of emamectin (1.5 g/ha) and abamectin (200 g/ha) were needed to effectively reduced damage caused by *M. incognita* on tomato using in-furrow application [7]. In the field trials, Milbeknock^®^ was applied by drip irrigation at 1.5 to 2.5 L/ha, equivalent to 28 and 47 g/ha of a.i (active ingredient), which may be insufficient for an effective nematode control. Certainly, those doses were lower than the effective dosage reported for abamectin.

Yield losses caused by RKN mainly depends on the initial population density of the nematode in soil before planting. Yield increases in response to the reductions in nematode densities at planting (Pi) were not observed in this study. This was attributed to the low *M. incognita* levels in the field plots (0.26 ± 0.03 J2/g soil), that were under the tolerance limits reported for the tomato (0.33-4 eggs + J2/g soil) [14]. Furthermore, it has been suggested that macrocyclic lactones have a rapid degradation rate in natural conditions so that their biological activity may be limited in some fields, and also that soil texture may influence their effectiveness in field applications [6]. The effective dosage of milbemectin for field application needs to be determined in various agro-environmental conditions and optimize according to the delivery system.

## 4. Material and Methods

### 4.1. Milbemectin

Pure milbemectin (>95%) was obtained from Cayman Chemical Europe OÜ, as a mixture of milbemycin A3 (30%) and milbemycin A4 (70%). Decreasing concentrations of milbemectin (30.0, 15.0, 7.0, 3.0, 1.5, 0.7, 0.3 and 0.0 μg/mL) were prepared by dilution in acetone: distilled water (5:95 *v*/*v*) + 0.5% Tween20 for the in vitro experiments. The solvent itself, without milbemectin, was used for untreated controls.

The commercial product Milbeknock^®^, containing 0.93% of milbemectin (*p/v*) as active ingredient, was provided by Belchim Crop Protection Spain. Other compounds included in the formulate as excipients (*w/w*) were white mineral oil (25%), cyclohexanone (25%), C9 aromatic hydrocarbons (25%), polyoxyethylene mono oleate mixture (10%), methyl laurate (fatty acid ester) (5%), octabenzone (1%) and 2,6-di-tert-butyl-p-cresol (1%). Six dilutions from Milbeknock^®^ were prepared in distilled water, containing 27.90, 13.95, 6.98, 2.79, 0.28 and 0.00 μg/mL of milbemectin, for the in vitro and in planta experiments. For the field trials, the product was applied diluted in water at the dosages shown in Table 4.

### 4.2. Nematode Inoculum

A population of *Meloidogyne javanica* (code MJ05) was used for the in vitro and in planta experiments. Plants of tomato ‘Roma’ (*Solanum lycopersicum*) were inoculated with the nematode and maintained for 8 weeks in a growth chamber at 25 °C to allow the nematode multiplication. Nematode eggs were extracted from the infected tomato roots by stirring them in a 0.5% NaOCl solution [15]. The egg suspension was concentrated on a 20 μm sieve and placed on Baermann funnels to obtain second-stage juveniles (J2). Juveniles hatching within 48 h were used as inoculum.

### 4.3. Effect of Milbemectin on Egg Hatching

Tests were conducted in vitro using 5 cm diameter Petri plates containing 10 mL of the respective dilutions of pure milbemectin and Milbeknock^®^. Micro-sieves with a 40 μm pore nylon mesh (BD falcon cell-strainer: BD Biosciences Discovery Labware, USA), were placed into each plate and 2–3 RKN egg masses were laid on each micro-sieve. The egg masses were then submerged into the respective dilutions of milbemectin or Milbeknock^®^. Plates were covered with their lids to prevent evaporation of the product, randomly located on a tray, and maintained at 25–27 °C in the dark. The effects of the treatments on egg hatching were assessed after 72 and 240 h incubation, by counting the number of juveniles hatched from the eggs. After readings, the sieves with the egg masses were placed into new Petri plates with tap water, incubated for additional 48 h at 25–27 °C in the dark. The numbers of juveniles per plate were counted again to determine if the effects of milbemectin on egg hatching were transient or permanent. At the end of the test, the egg masses from each plate were recovered and placed in an Eppendorf tube and the remaining eggs were released by maceration in 1 mL of 0.5% NaOCl solution. Non-hatched eggs were counted and considered as non-viable or dead. The egg mortality (EM) caused by the treatments was corrected by eliminating the natural mortality given by the untreated controls, according to Schneider–Orelli formula [16] (1).

(1) $$EMx=\frac{emx-emc}{1-emc}\times 100$$

*emx*: (non-hatched eggs in treatment x)/(total number of eggs treatment x).

*emc*: (non-hatched eggs in the untreated control) / (total number of eggs in the untreated control).

Each treatment was repeated four times and the test was carried out twice.

### 4.4. Effects of Milbemectin on Juvenile Motility

Approximately 100 second-stage juveniles (J2) were added in 20 μL of water to each well of a 24 multi-well plate containing 3 mL of the respective dilutions of milbemectin or Milbeknock^®^. The plates were incubated at 25–27 °C in the dark. The effects of the treatments on juvenile motility were determined after 24 h incubation, by counting the numbers of motile and immotile juveniles per well. At the end of the test, juveniles present in each well were recovered by passing the juvenile suspension through a 20 μm aperture sieve and washing with distilled water to eliminate any trace of the products. The recovered juveniles were placed in a new multi-well plate containing 3 mL of tap water and incubated for additional 24 h at 25–27 °C in the dark to determine if the effect of the product on juvenile motility was transient or permanent. The numbers of motile and immotile juveniles were counted again. Immotile juveniles were considered dead when they did not move on probing with a fine needle. The J2 mortality (JM) caused by the treatments was corrected by eliminating the natural mortality given by the untreated controls, according to Schneider–Orelli formula [16] (2).

(2) $$JMx=\frac{jme-jmc}{1-jmc}\times 100$$

*jme*: (immotile juveniles in treatment x)/(total juveniles in treatment x).

*jmc*: (immotile juveniles in untreated control)/(total de juveniles in untreated control).

Each treatment was repeated four times and the test was carried out twice.

### 4.5. Effects of Milbeknock^®^ on Nematode Root Infection

Two pot experiments were established in a growth chamber with three treatments each: (i) soil inoculated with RKN, (ii) soil inoculated with RKN and treated with Milbeknock^®^ and (iii) non-inoculated and untreated control. Treatments were replicated six and five times in experiments 1 and 2, respectively. Pots of 1250 cm^3^ capacity were filled with a 1000 g of a potting mix (vermiculite) and watered to field capacity. Soil was infested 24 h later by adding aliquots of a nematode suspension into nine holes made in the soil at 5 cm depth. Nematode inoculum consisted of 1250 and 2500 nematodes per plant in the first and second experiment, respectively. Treatments were applied 24 h after infesting the soil with the nematode; 0.3 mL of Milbeknock^®^ diluted in 300 mL of water was added per pot (2.8 mg of milbemectin per pot) as a soil drench. The untreated pots, with or without the nematodes, received the same amount of water but without Milbeknock^®^. Tomato ‘Roma’ seedlings were transplanted within the following 24h. Plants were maintained in a growth chamber at 25–27 °C, watered daily as needed and fertilized with a slow-release fertilizer (15% N + 10% P_2_O_5_ + 12% K_2_O + 2% MgO_2_ + microelements) (Osmocote^®^, Scotts Company LLC) by adding 2 g per pot at the beginning of the experiments.

Experiments were terminated 47 days after transplanting the tomato plants. Plant tops were cut at ground level and their length and fresh and dry top weight determined. Tops were desiccated in an oven at 60 °C for 48 h. Roots were separated carefully from soil and weighed. Egg masses were counted in root subsamples of 5 g per plant. To facilitate the counting of egg masses, roots were stained with a 0.1 g/L erioglaucine solution (Aldrich Chemical Co. Inc.) for 2 h [17].

### 4.6. Effects of Milbeknock^®^ on Root-Knot Nematode Disease in Field Trials

The study was carried out in an experimental field (poly-tunnel unheated plastic house) of 1000 m2 located at the IFAPA Centre in Chipiona, Cadiz, Spain (36°44′56′’N 6°24′06′’W), which is regularly used for the cultivation of vegetables. The soil was sandy (84% sand: 10% silt: 6% clay), pH 7.7, electrical conductivity 3.8 dS/m and 0.5% organic matter and it was naturally infested with a population of *Meloidogyne incognita*. Field soil was carefully ploughed longitudinally and transversally for homogenization, divided into sixteen elementary plots of 28.5 m^2^ (9.5 × 3 m) each and irrigated to field capacity. Two days later, nematode pre-treatment population densities were determined by soil sampling of each elementary plot (P0). Composite soil samples consisted of ten soil cores distributed over the area of each plot and they were collected with an Auger sampling tool (2.5 cm diameter, 30 cm depth). Nematodes were extracted from two subsamples of 250 g of soil, using the centrifugation method [18].

Four treatments were applied to soil including an untreated control and the commercial product based on abamectin Tervigo^®^ (Syngenta Crop Protection AG) for comparison (Table 1). The experimental design was a randomized complete block design with four replicates per treatment. The whole trial was repeated the following year in the same site. The products were applied before and two weeks after planting tomato through an irrigation system with a water flow of 4.4 L/h for approximately 3 h 15 min (5000 L/ha). Forty-eight hours after soil treatments, ten plants of tomato ‘Amapola’ were planted per plot, in two rows of five plants each, with a distance between plants 1 m. At planting, a second soil sampling was performed to determine nematode population densities after the treatments in each plot (Pi). Composite soil samples were collected and processed as described above. Nematode mortality in each plot was calculated according to the formula (3).

(3) $$nm=1-\frac{P_{i}}{P_{0}}$$

Nematode mortality (NM) caused by each treatment was corrected by eliminating the natural mortality given by the untreated controls, according to Schneider–Orelli formula [16] (4).

(4) $$NMx=\frac{nmx-nmc}{1-nmc}\times 100$$

*nmx*: (nematode mortality in treatment x).

*nmc*: (nematode mortality in untreated control).

Plots were watered as needed and fertilized weekly with a solution NPK (15-5-30) at 31 kg per ha and iron and micronutrients at 0.9 kg per ha. For pest control, yellow and blue chromotropic and pheromone traps were placed within the plastic house. Weeds were removed manually during the growing cycle. Fruits produced per plant were harvested once per fortnight, and the number of fruits and total fruit weight values recorded and cumulated yield throughout the growing cycle expressed per plant. The growing cycle lasted 117 days. Plants were cut at ground level, root systems were ripped off, and the root gall indices were determined according to a scale 0–10 [19].

### 4.7. Statistical Analyses

Data are expressed as mean ± standard error and were analyzed with the Statgraphics Centurion XVI^®^ (Statpoint Technologies Inc., Warrenton, VA, USA) statistical software. The Kolmogorov–Smirnov and Brown–Forsythe tests were applied to data to check for normality and homoscedasticity of variances; if significant, data were arcsine-transformed and subjected to the same tests once more. When normality and homoscedasticity of variances could be assumed, data were analyzed by ANOVA. If F values were significant, the means were compared by the HSD Tukey test (*p* < 0.05). When the homoscedasticity of variances could not be assumed, Welch’s ANOVA was used. When normality was not reached after transformation, the data were analyzed by Kruskal-Wallis non-parametric tests, and if H values were significant, means were compared by Dunn’s multiple comparison test (*p* < 0.05).

Dose–response probabilistic models (PROBIT) were used on corrected egg and J2 mortality data to determine the lethal concentration (LC) of milbemectin that inhibit nematode egg hatching and J2 motility by 50 and 90% (LC50 and LC90).

## 5. Conclusions

Milbemectin shows high efficacy against root-knot nematodes in vitro and in planta. Milbemectin immobilized 90% of RKN juveniles and reduced egg hatching by 50% at 30 μg/mL concentration. Increasing concentrations and exposures times improved the efficacy of milbemectin in reducing RKN J2 motility and egg hatching. Milbemectin significantly reduces reduced RKN soil population densities in a range of 50–60%. This biopesticide shows a great potential to be formulated as an effective nematicide.

## Figures and Tables

**Table 1:** Effect of Milbeknock^®^ on root infection by *Meloidogyne javanica* on tomato ‘Roma’ in pot experiments 47 days after transplanting.

Experiment	Nematode Inoculum	Treatment	Egg Masses/Plant
1	1250 J2/plant	Milbeknock^®^	4 ± 1 a
		Untreated	296 ± 24 b
2	2500 (eggs + J2)/plant	Milbeknock^®^	13 ± 4 a
		Untreated	746 ± 29 b

Data are mean of six and five replicates ± standard error of the means in experiment 1 and 2, respectively. Data followed by the same letter within the same experiment do not differ significantly by the Dunn test (*p* < 0.05).

**Table 2:** Effects of Milbeknock^®^ on growth parameters of tomato ‘Roma’ infected by *Meloidogyne javanica* in pot experiments 47 days after transplanting tomato.

Experiment	Treatment	Shoot Length (cm)	Fresh Top Weight (g)	Dry Top Weight (g)	Fresh Root Weight (g)
1	Non RKN	72.8 ± 1.5 a	70.6 ± 5.5 a	8.9 ± 1.0 a	9.7 ± 0.5 a
	RKN untreated	76.1 ± 3.3 a	73.4 ± 5.5 a	9.6 ± 0.7 a	11.5 ± 0.7 a
	RKN Milbeknock^®^	75.3 ± 2.6 a	63.8 ± 2.2 a	8.4 ± 0.8 a	13.4 ± 1.0 a
2	Non RKN	66.6 ± 3.5 a	80.1 ± 4.1 a	11.2 ± 0.5 a	21.1 ± 0.9 a
	RKN untreated	59.8 ± 0.6 a	86.6 ± 6.3 a	11.8 ± 0.7 a	28.5 ± 1.3 a
	RKN Milbeknock^®^	67.6 ± 2.7 a	81.9 ± 3.1 a	11.6 ± 0.6 a	28.1 ± 2.0 a

Data are mean of six and five replicates ± standard error of the means in experiment 1 and 2, respectively. Data followed by the same letter within the same column and experiment do not differ significantly by the Dunn test (*p* < 0.05). Root-knot nematodes, RKN.

**Table 3:** Effects of abamectin and milbemectin soil treatments on mortality of *Meloidogyne incognita* soil populations and growth of tomato ‘Amapola’ in field trials.

Treatment	Nematode Mortality (%)	Gall Index	Yield per Plant	
			No. Fruits	Fruit Weight (g)
Untreated control	-	5.5 ± 1.0 a	34 ± 3 b	5411 ± 550 a
Tervigo^®^ (5.0 L/ha)	61 ± 2.3 a	4.6 ± 0.5 a	41 ± 3 a	6010 ± 523 a
Milbeknock^®^ (2.5 L/ha)	59 ± 1.9 a	4.9 ± 0.6 a	36 ± 2 b	5380 ± 525 a
Milbeknock^®^ (1.5 L/ha)	52 ± 1.6 b	5.0 ± 0.9 a	34 ± 2 b	5112 ± 497 a

Data are the mean ± standard error of 80 replicates (10 plants × 4 plots × 2 trials). Data followed by the same letter within the same column do not differ significantly by the HSD Tukey test (*p* < 0.05).

**Table 4:** Treatments applied to soil infested with *Meloidogyne incognita* in field trials.

Trade Name	Active Ingredient	Dosage	Applications
Untreated	-		
Tervigo^®^	Abamectin 1,67%	5.0 L/ha	2
Milbeknock^®^	Milbemectin 0,93%	2.5 L/ha	2
Milbeknock^®^	Milbemectin 0,93%	1.5 L/ha	2
